# Polypoid Lymphangiectasia in the Sigmoid Colon: A Case Report of a Rare Entity

**DOI:** 10.7759/cureus.40632

**Published:** 2023-06-19

**Authors:** Anwar Alshaakh Moh’D Mari, Aarushi Varshney, Kristin Recker, Jignesh Parikh, Vania Zayat

**Affiliations:** 1 Internal Medicine, University of Central Florida HCA Healthcare GME, Orlando, USA; 2 Pathology, University of Central Florida College of Medicine, Orlando, USA; 3 Pathology, Orlando Veterans Affairs Medical Center, Orlando, USA

**Keywords:** lymphatic, polypoid lesion, intestinal lymphangiectasia, chronic abdominal pain, sigmoid colon

## Abstract

Intestinal polypoid lymphangiectasia is an uncommon disorder involving an improperly formed enteric lymphatic system. It is characterized by lymphatic vessel dilatation with impaired drainage or obstruction of the lymph from the intestine. In this report, we present a case of a 73-year-old male patient with chronic intermittent left lower quadrant abdominal pain for one year who was found to have a sigmoid colon polyp on a colonoscopy. Upon microscopic examination, the polyp revealed dilated lymphatic vessels staining strongly for D2-40 (lymphatic vessel marker), supporting the diagnosis of polypoid lymphangiectasia. Intestinal lymphangiectasia has a broad differential diagnosis, warranting histopathological examination for a definitive diagnosis.­­­­­­­­­­­­­­­

## Introduction

Intestinal polypoid lymphangiectasia is an uncommon disorder characterized by an improperly formed enteric lymphatic system with a mass lesion protruding from the mucosal surface into the intestinal lumen [1]. It involves lymphatic vessel dilatation with impaired lymph drainage or obstruction of the lymph flow from the intestine. A recent review of the literature identified a total of 49 cases of primary intestinal lymphangiectasias between 1993 and 2020 with the duodenum being the most frequently reported site [2]. According to a retrospective evaluation of 1,866 consecutive endoscopic examinations between January 2005 and June 2006, duodenal lymphangiectasia occurred in 1.9% of cases [3]. Clinically, patients may present with protein-losing enteropathy (PLE), hypoproteinemic edema, chylous ascites, pleural effusion, acute appendicitis, recurrent diarrhea, and intestinal obstruction [2,4]. On colonoscopy, lymphangiectasia presents either as whitish spots or specks, or, on endoscopy, as yellowish, well-circumscribed, raised mucosal or submucosal lesions [4,5]. Patients with gastrointestinal vascular and lymphatic malformation may seldom suffer from the presence of polypoid [6]. Polypoid vascular and lymphatic malformation of the small intestine is rare [6,7]. To our knowledge, there have been no reported cases involving the sigmoid colon. We present a case of intestinal lymphangiectasia with an unusual presentation as a polyp in a rare location: the sigmoid colon. Given the nonspecific clinical and colonoscopic presentation of our case, a histopathological examination was warranted for a definitive diagnosis.

## Case presentation

A 73-year-old male presented to the primary care clinic for his regular yearly check-up. The patient complained of severe intermittent non-radiating abdominal pain localized to the left lower quadrant for a duration of one year. He also had associated occasional constipation. There were no complaints of nausea, vomiting, weight loss, hematochezia, or melena. No specific triggers were identified. His pain was not associated with food intake or bowel movement. The patient had a significant medical history of hypertension, hypogonadism, non-insulin-dependent diabetes, benign prostatic hyperplasia, and gastroesophageal reflux disease (GERD). No colonoscopy had been performed previously, and no family history of inflammatory bowel disease or colon cancer was reported. An abdominal exam showed a soft, non-distended, non-tender abdomen with normoactive bowel sounds. With regard to lower extremities, no peripheral edema was noted.

**Figure 1:**
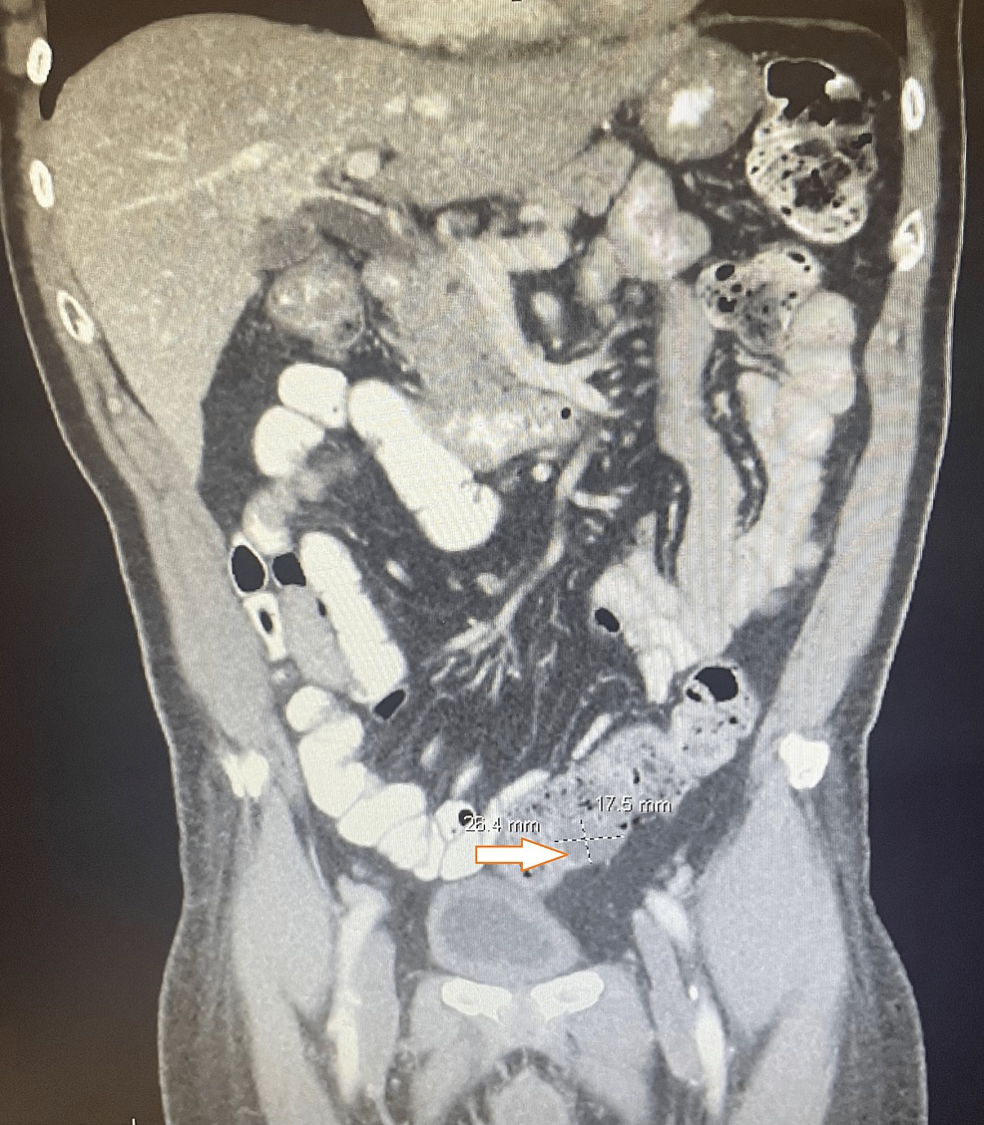
CT scan of the abdomen and pelvis Triangular soft tissue opacity in the sigmoid colon measuring about 26 x 18 mm on coronal image CT: computed tomography

The patient underwent a blood examination, which showed a total protein of 7.8 g/dL and albumin of 3.7 g/dL. His lipid profile, which included triglycerides, LDL, and HDL, was within the normal range. A CT scan of the abdomen and pelvis was performed, which showed a triangular soft tissue opacity in the sigmoid colon measuring about 26 x 18 mm on the coronal image, with diffuse colonic diverticula, without any convincing evidence of focal acute diverticulitis (Figure 1). There was no evidence of intramural abscess or bowel obstruction.

**Figure 2:**
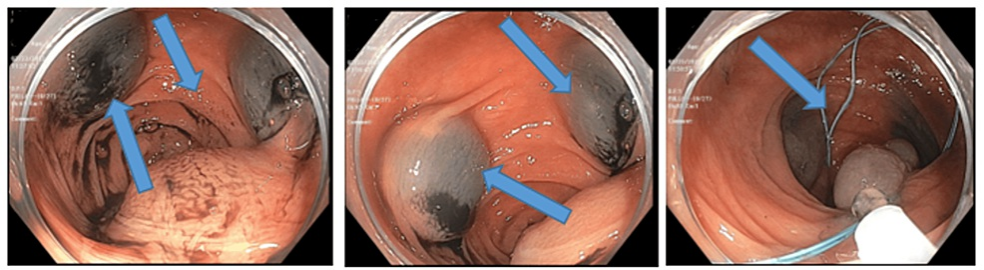
Colonoscopy showing multiple polyps in the sigmoid colon

**Figure 3:**
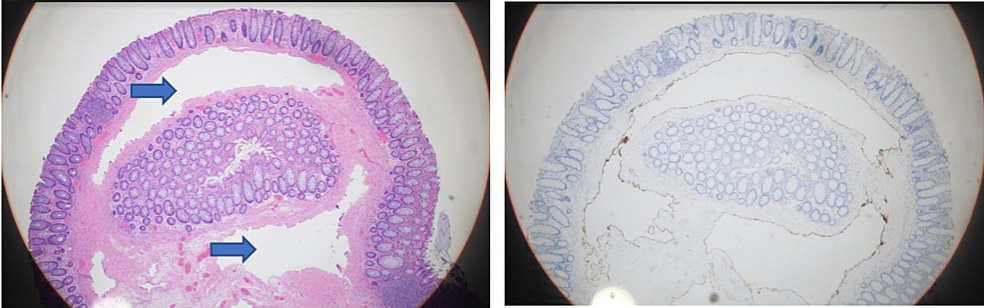
(a) The sigmoid polypectomy microscopically with H&E stain; the arrows point at the lymphangiectasia (4X magnification). (b) D2-40 stain highlighting the lymphatic lining (4X magnification)

The patient subsequently underwent a colonoscopy, which showed multiple polyps in the colon (ascending, transverse, and sigmoid colon) (Figure 2). Two sessile polyps measuring 3 mm each were found in the ascending colon. A 3-mm sessile polyp was found in the transverse colon, and a 25-mm submucosal polypoid lesion was found in the sigmoid colon. Microscopic examination of the polyps in the ascending and transverse showed fragmented colonic mucosa with focal hyperplastic changes. The sigmoid polypectomy microscopically was composed of submucosal dilated vessels (Figure 3a), which were evaluated further with D2-40 (lymphatic vessel marker) stain. The D2-40 stain (Figure 3b) highlighted lymphatic vessels in the colon polyp. The CD34 stain was applied and showed weak positivity. While CD34 staining was weakly positive, the protein can be present in lymphatic vascular endothelium occasionally. However endothelial cells in arteries and veins are always negative for D2-40, while CD34 can be occasionally and irregularly expressed in lymphatic endothelium with weak staining like in our case [8]. The presence of the strongly positive lymphatic specific marker D2-40 and weakly positive CD34 helped establish our diagnosis.

## Discussion

Lymphangiectasia is a rare pathological dilation of lymphatics seen in the intestines and hence termed “intestinal lymphangiectasia''. It can impair the lymph flow, leading to obstruction from enlargement, and can lead to loss of protein [2]. In children, it manifests as primary lymphatic malformations and is termed lymphangioma. In adults, it presents as dilation of pre-existing lymphatics, termed lymphangiectasia. It is essential to further evaluate these findings if detected, as they may be secondary to an underlying malignancy [5]. Lymphangiectasia is most commonly seen in the small intestine with very few reported cases of it being found in the colon. In general, duodenal lymphangiectasia is a rare finding seen incidentally in approximately 1.9% of the adult population who undergo endoscopy [3]. It is usually characterized by white spots and yellow-white mucosal or submucosal nodules on endoscopy, and the presence of a polypoid lesion is extremely rare [7].

In our case, the lesion was present in the colon, as opposed to the more common location of the small intestine. In addition, instead of the commonly seen white spots/specks on endoscopy, our case presented as a polypoid lesion. The sigmoid polypectomy microscopically was composed of submucosal dilated vessels, which were evaluated further with D2-40 (lymphatic vessel marker) stain. The D2-40 stain highlighted that the vessels were lymphatic vessels with a lack of surrounding endothelial lining or smooth muscles. These findings helped confirm the diagnosis of polypoid intestinal lymphangiectasia.

It is vital to further investigate such a rare finding in this patient population in order to rule out underlying etiologies such as Waldenström macroglobulinemia, inflammatory etiologies, sarcoidosis, or other mass-forming intestinal malignancies [5]. Other differential diagnoses can include PLEs, which are associated with lymphangiectasia secondary to constrictive pericarditis, Whipple’s disease, Crohn’s disease, intestinal tuberculosis, and HIV-related enteropathy [5], These conditions have the potential to impact the gastrointestinal tract, resulting in inflammation, structural abnormalities within the intestines, and the potential manifestation of polypoid growth patterns. Upon review of the patient’s history and physical exam, there was no evidence of an underlying etiology or any of the risk factors mentioned above. Following the procedure, the patient experienced a positive outcome and underwent regular six-month follow-ups under the care of his primary physician. He was advised to undergo a repeat colonoscopy in three years. In addition, it would be beneficial to follow up with gastroenterologists to check for the development of malignancies such as lymphoma transformation, gastrointestinal bleeding, or other potential complications.

Diagnostic testing for lymphangiectasia usually involves intestinal endoscopy and biopsy with histological evaluation. Histological evaluation is warranted to arrive at the final diagnosis as it helps determine if it is lymphatic-based or not. If endoscopic visualization and biopsy fail to confirm a diagnosis, lymphangiography may also be utilized in the diagnostic workup.

The management of lymphangiectasia appears to be similar regardless of whether the cases are found in the small intestine or colon. Treatment with a strict low-fat diet and supplemental medium-chain triglycerides is necessary for all patients with this condition [1]. Medium-chain fatty acids are a critical component as they bypass the lymphatics and go directly into the portal venous system [1]. Clinical and biochemical findings like hypoalbuminemia can recur in patients non-adherent to a low-fat diet. Other treatments include antiplasmin, octreotide, corticosteroids, albumin infusion, and surgery [1].

Literature on the outcomes of intestinal lymphangiectasia is currently limited due to the uncommon nature of the disease. However, one literature review study in 2010 found that dietary intervention is effective in a majority of cases (around 63%). Additionally, they reported malignant transformation of lymphoma in 5% of patients ranging in age between 19 and 45 years after primary intestinal lymphangiectasia onset [9]. Further research is needed to gain more insights into this entity, especially relating to its management and long-term outcomes.

## Conclusions

Intestinal polypoid lymphangiectasia is an uncommon disorder that requires imaging, laboratory assessment, and histological evaluation for a definitive diagnosis. We discussed a unique case presenting as a polyp in the sigmoid colon instead of the common presentation of white spots in the small intestine. A diagnosis of intestinal lymphangiectasia warrants further evaluation to rule out underlying etiologies such as Waldenström macroglobulinemia, inflammatory disease, sarcoidosis, and malignancy. In addition, raising awareness about this disorder is essential since patients are at risk of gastrointestinal bleeding and other comorbidities associated with PLE.
